# Metabolomics and lipidomics in *Caenorhabditis* *elegans* using a single-sample preparation

**DOI:** 10.1242/dmm.047746

**Published:** 2021-04-27

**Authors:** Marte Molenaars, Bauke V. Schomakers, Hyung L. Elfrink, Arwen W. Gao, Martin A. T. Vervaart, Mia L. Pras-Raves, Angela C. Luyf, Reuben L. Smith, Mark G. Sterken, Jan E. Kammenga, Antoine H. C. van Kampen, Georges E. Janssens, Frédéric M. Vaz, Michel van Weeghel, Riekelt H. Houtkooper

**Affiliations:** 1 Laboratory Genetic Metabolic Diseases, Amsterdam UMC, University of Amsterdam, Meibergdreef 9, 1105 AZ Amsterdam, The Netherlands; 2 Core Facility Metabolomics, Amsterdam UMC, University of Amsterdam, Meibergdreef 9, 1105 AZ Amsterdam, The Netherlands; 3 Bioinformatics Laboratory, Amsterdam Public Health Research Institute, Amsterdam UMC, University of Amsterdam, Meibergdreef 9, 1105 AZ Amsterdam, The Netherlands; 4 Laboratory of Nematology, Wageningen University and Research, Droevendaalsesteeg 1, 6708 PB Wageningen, The Netherlands

**Keywords:** *C. elegans*, Lipidomics, Metabolism, Metabolomics

## Abstract

Comprehensive metabolomic and lipidomic mass spectrometry methods are in increasing demand; for instance, in research related to nutrition and aging. The nematode *Caenorhabditis* *elegans* is a key model organism in these fields, owing to the large repository of available *C. elegans* mutants and their convenient natural lifespan. Here, we describe a robust and sensitive analytical method for the semi-quantitative analysis of >100 polar (metabolomics) and >1000 apolar (lipidomics) metabolites in *C. elegans*, using a single-sample preparation. Our method is capable of reliably detecting a wide variety of biologically relevant metabolic aberrations in, for example, glycolysis and the tricarboxylic acid cycle, pyrimidine metabolism and complex lipid biosynthesis. In conclusion, we provide a powerful analytical tool that maximizes metabolic data yield from a single sample.

This article has an associated First Person interview with the joint first authors of the paper.

## Abstract (precis)

**Summary:** We describe an integrated metabolomics and lipidomics method for analyzing metabolites in *C. elegans* using a single-sample preparation, which provides a robust way of obtaining comprehensive, high-quality metabolic data from biological samples.

## INTRODUCTION

Considerable advances in high-performance liquid chromatography (HPLC), mass spectrometry (MS) and nuclear magnetic resonance (NMR) make it possible to reliably detect tens of thousands of compounds (Johnson et al., 2016). Additionally, semi-automatic annotation of metabolites and data analysis tools have greatly improved the quality and robustness of metabolomic platforms, allowing for an improved sample throughput and ease of data analysis and interpretation (Haug et al., 2017). As a consequence, metabolomic analysis has seen a surge in popularity over the past decades, and the importance and intricacies of metabolism in health and disease are becoming increasingly evident (Haug et al., 2017). In turn, this has prompted increased demand for reliable and robust metabolomic methods for polar and apolar metabolite analyses in model organisms and human tissues (Edison et al., 2016).

For many years, *Caenorhabditis elegans* nematodes have been used intensively to investigate genetics, development, as well as aging. *C. elegans* is a versatile model system as genetic influences can be tested with relative ease, owing to the availability of large repositories of mutants as well as RNA interference (RNAi) libraries. Moreover, genetic reference populations have been generated for *C. elegans* in which natural genetic variation is present at a level similar to that in the human population (Thompson et al., 2015). This way, meaningful data on population genetics and gene-by-environment interactions can be obtained using, for instance, dietary interventions (Watson et al., 2013; Doroszuk et al., 2009). More recently, *C. elegans* has become a relevant model to investigate metabolism, because metabolism was identified as a key regulator of traits such as aging (Maglioni and Ventura, 2016; Gao et al., 2018; Houtkooper et al., 2010a,b). Metabolic network models for *C. elegans* were recently constructed (Yilmaz and Walhout, 2016; Gebauer et al., 2016), and a curated consensus is currently being assembled in a European-led consortium (Witting et al., 2018). The success of such endeavors relies heavily on accurate and robust metabolomics methods (Reed et al., 2017).

Metabolite measurements in mammalian tissues are commonplace (Johnson et al., 2016; Herzog et al., 2016; Newgard, 2017); however, in *C. elegans* they are sparsely applied (Witting and Schmitt-Kopplin, 2016). Methods for *C. elegans* metabolite analyses are predominantly based on gas chromatography (GC)-MS (Perez and Van Gilst, 2008; Watts and Ristow, 2017) and NMR spectroscopy (Witting and Schmitt-Kopplin, 2016; Watts and Ristow, 2017; Yang et al., 2006). Drawbacks of these approaches include the need for large quantities of worms and a limited number of metabolites that can be quantified (Table 1). Recent developments using targeted metabolomics with liquid chromatography (LC)-MS allow the measurement of hundreds of metabolites, including fatty acids and amino acids, in a sample of ∼2000 worms (Gao, 2017; Hastings et al., 2019).

**Table 1.**
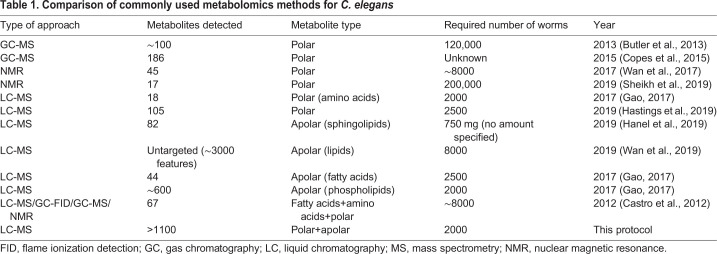
Comparison of commonly used metabolomics methods for *C. elegans*

Although these methods are useful when focusing on specific metabolite classes, they rely on separate extraction procedures for both polar and apolar metabolites in biological replicates, making them less suitable for screening purposes. Previously, an integrated omics method employing both LC-MS/MS and GC-MS using derivatizations was used on bacteria, urine, cell cultures, mouse tissue and plant leaves to detect 51 polar metabolites and 84 lipid species (Nakayasu et al., 2016). Hence, we set out to develop a comprehensive omics protocol for *C. elegans*, combining our previously reported metabolomics and lipidomics methods into a unified extraction, a variation of the popular Folch or Bligh and Dyer liquid-liquid extraction (LLE) method (Bligh and Dyer, 1959), and reliant exclusively on LC-MS for analysis (Molenaars et al., 2020; Held et al., 2020).

This type of LLE is a user-friendly and highly effective laboratory staple, capable of isolating both the metabolome and lipidome of *C. elegans*. This quality is not a given for extractions, as endogenous metabolites span a wide range of physicochemical properties, making it difficult to extract a large range of the metabolome with a single solvent. Additionally, polar solvents typically lack the ability to precipitate interfering proteins in biological samples, making a simple water extraction of polar metabolites impractical. An elegant way to remedy these issues is to use an LLE like the Bligh and Dyer method. In this case, a highly apolar solvent, e.g. chloroform, is used to precipitate protein and facilitate the breakdown of biological organization, while a polar solvent is added to extract polar metabolites in a separate layer. This type of extraction also doubles as a separation step, removing apolar compounds from the polar layer (and vice versa), thereby reducing ion suppression effects during MS analysis. Interestingly, this type of two-phase extraction was first applied for extracting lipids in the (predominantly) chloroform phase (Bligh and Dyer, 1959). We used such a two-phase extraction on *C. elegans* to perform both metabolomics and lipidomics in a single sample.

The method presented here provides a detailed step-by-step protocol for sample collection and processing, metabolite extraction, annotation and relative quantification in *C. elegans.* We demonstrate that metabolomic and lipidomic analysis can be performed on a single sample using a single extraction protocol, reducing sample preparation and throughput time without compromising metabolite identification. Additional benefits include a reduction in the required number of *C. elegans* cultures, as well as a significant reduction in waste. With this protocol, we can semi-quantitatively measure >100 polar and >1000 apolar metabolites, from all major metabolite classes in a sample of ∼2000 worms. Moreover, this method can be easily adapted for other model systems, cells and tissues.

## RESULTS

### Validation of polar metabolite (metabolomics) analysis in *C. elegans*

**Fig. 1.**
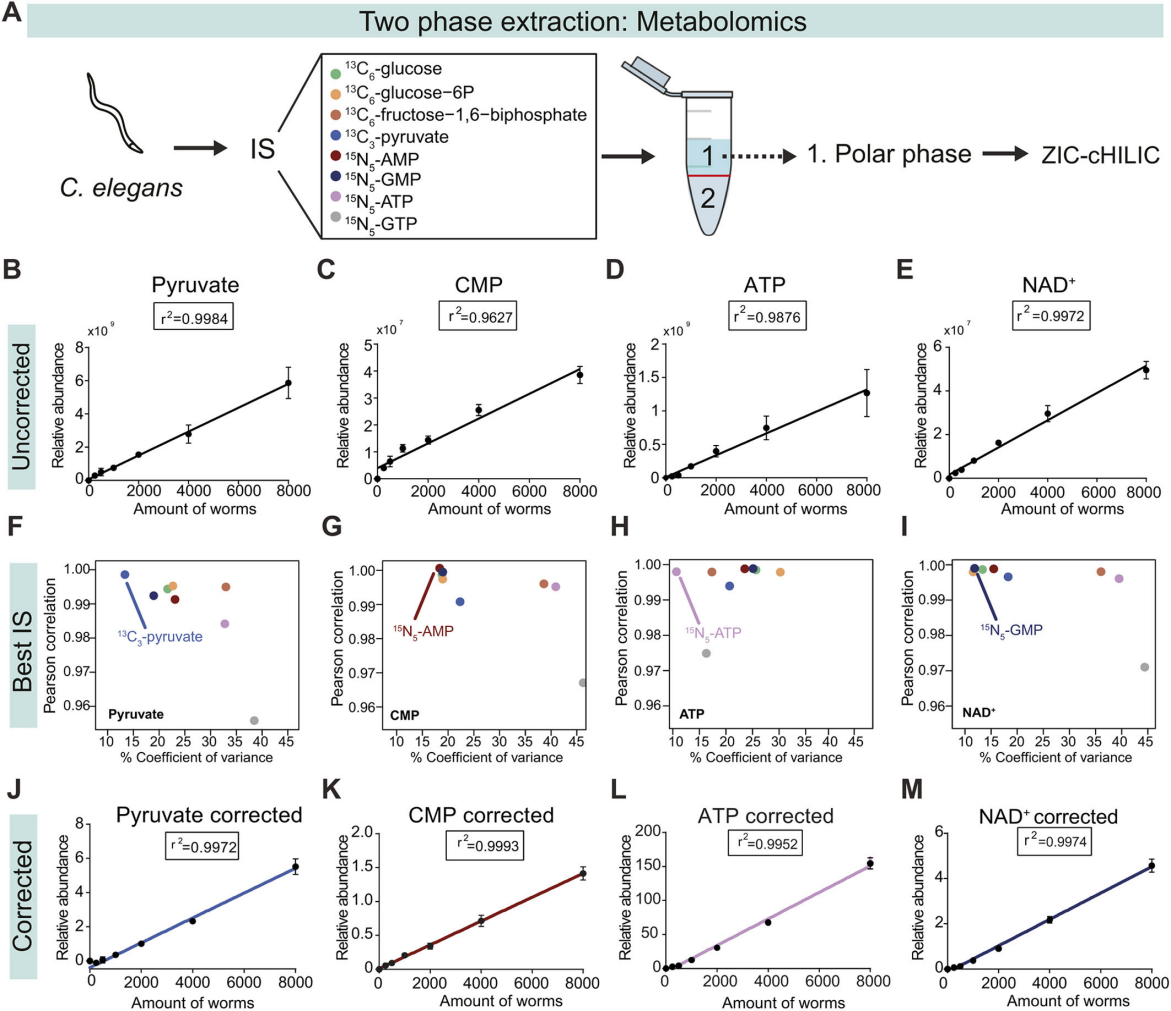
**Validation and linearity of metabolites extracted from polar phase.** (A) Internal standard (IS) was added to *C. elegans* pellet and, using a two-phase extraction, the upper polar phase was processed for ZIC-cHILIC. (B-E) Linearity of four example metabolites – pyruvate (B), CMP (C), ATP (D) and NAD^+^ (E) – shows *r*^2^ >0.98. (F-I) For pyruvate (F), CMP (G), ATP (H) and NAD^+^ (I), the best IS was determined per metabolite by plotting Pearson's correlation coefficient against coefficient of variance. (J-M) Linearity of pyruvate (J), CMP (K), ATP (L) and NAD^+^ (M) after correction for their best IS shows *r*^2^ >0.99. Data points represent mean±s.d. with *n*=4 biological replicates.

In order to enable validation, we used *C. elegans* pellets from biological replicates containing different numbers of worms and extracted polar metabolites from the upper phase of the LLE (Fig. 1A). Polar metabolites were separated using a variation of hydrophilic interaction LC (ZIC-cHILIC) and measured using a Q Exactive Plus Orbitrap mass spectrometer. For each of the annotated polar metabolites, we determined their linear response because loss of linearity of the MS response is a good measure of bias in sample preparation or analysis (Table S1). Four example metabolites – pyruvate, cytidine monophosphate (CMP), adenosine triphosphate (ATP) and nicotinamide adenine dinucleotide (NAD^+^) – showed strong linearity across the range of worms before applying normalization for internal standard (Fig. 1B-E; Table S1). We then established which internal standards to use for each metabolite (Table S2). Selection of the internal standard was based on the combination of the lowest coefficient of variation for biological replicates after correction with internal standard (analyte peak area divided by internal standard peak area), and highest Pearson correlation coefficient after correction across the number of worms (Fig. 1F-I). Applying these internal standards for the data normalization led to even better linearity for pyruvate, CMP, ATP and NAD^+^ (Fig. 1J-M; Table S2).

Altogether, we established optimal internal standard conditions for each of the polar metabolites and found that each of these can be reliably measured in a sample of ∼2000 worms. Subsequently, we determined the analytical repeatability of our method using a repeated injection of a pooled sample throughout the analytical run. For each metabolite measured in these samples, a relative standard deviation (RSD) was calculated (Table S1). We observed that 99% of polar metabolites had an RSD <30 between different extractions, while 97% of polar metabolites had an RSD <30 when repeatedly injecting a pool of those samples, indicating that our method is highly robust.

### Validation of the apolar metabolite (lipidomics) analysis

**Fig. 2.**
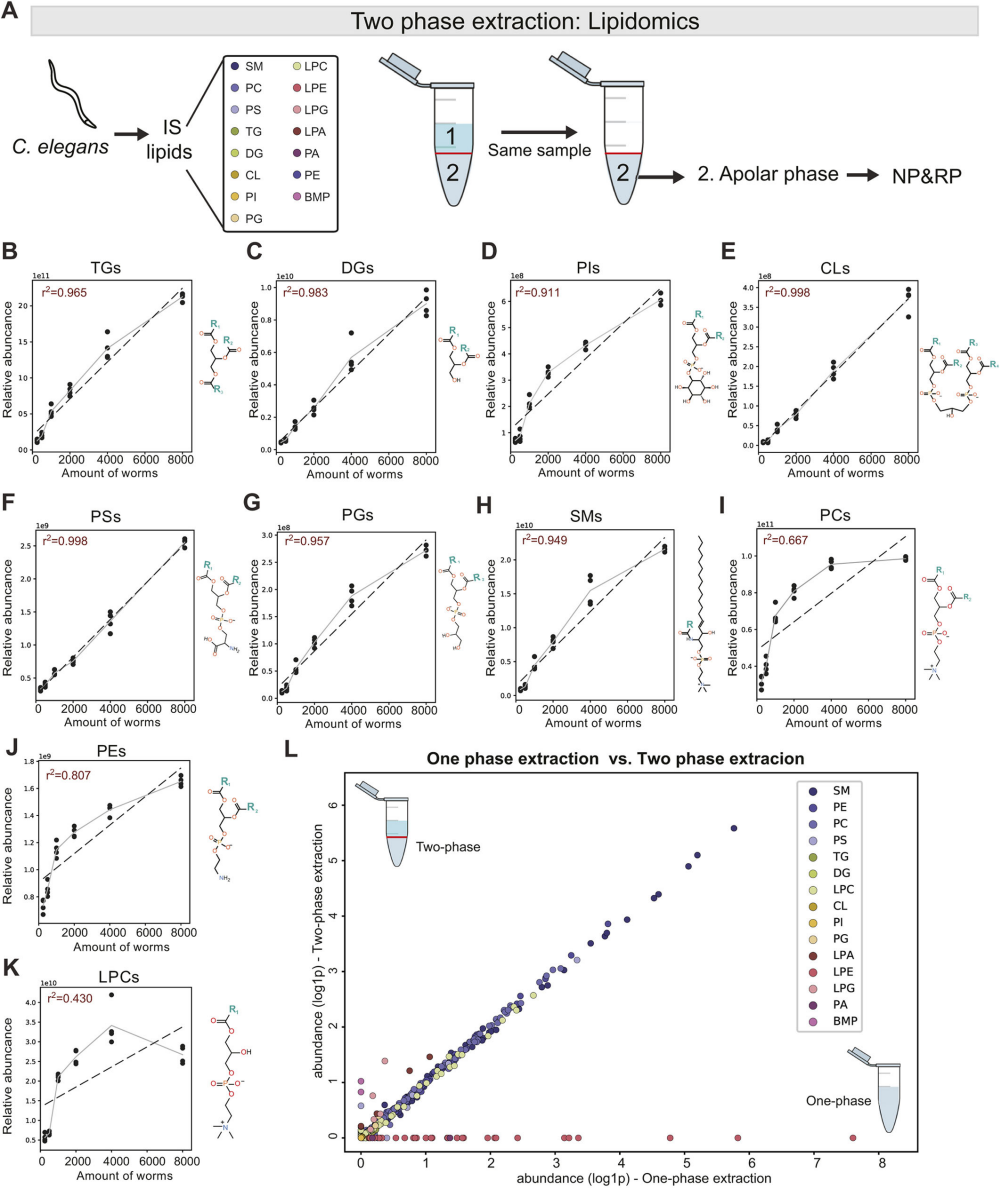
**Validation of lipids extracted from apolar phase and comparison of two-phase versus one-phase lipid extraction.** (A) IS was added to *C. elegans* pellet and, using a two-phase extraction, the lower apolar phase from the same sample was processed for normal-phase (NP) and reversed-phase (RP) chromatography. (B-K) Linearity of triacylglycerols (TGs; B), diacylglycerols (DGs; C), phosphatidylinositols (PIs; D), cardiolipins (CLs; E), phosphatidylserines (PSs; F), phosphatidylglycerols (PGs; G), sphingomyelins (SMs; H), phosphatidylcholines (PCs; I), phosphatidylethanolamines (PEs; J) and lysophosphatidylcholines (LPCs; K). (L) Comparison of relative abundances (log1p) from one-phase extraction versus two-phase extraction. Data points represent biological replicates with *n*=4. BMP, bis(monoacylglycero)phosphate; LPA, lysophosphatidic acid; LPE, lysophosphatidylethanolamine; LPG, lysophosphatidylglycerol; PA, phosphatidic acid.

Instead of a dedicated single-phase extraction we reported previously (Molenaars et al., 2020), we used the ‘left-over’ apolar phase from our two-phase extraction on biological replicates to analyze lipids (Fig. 2A; Table S3). Lipids were separated using both a normal-phase (NP) and reversed-phase (RP) chromatography method and measured on a Q Exactive Plus mass spectrometer. Additionally, a pooled sample was injected ten times.

The lipidome is an enormously diverse class of metabolites with widely varying polarities. For instance, at the apolar end of the spectrum, triacylglycerols (TGs) consist of a glycerol backbone and three fatty acid tails. Owing to these uniformly apolar qualities, TGs of any chain length partition almost exclusively to the chloroform phase during the two-phase extraction. This results in a linear relationship between the number of worms and the measured abundance of TGs (Fig. 2B). A similar pattern is observed for other major lipid classes containing multiple acyl side chains, such as diacylglycerols (DGs), phosphatidylinositols (PIs), cardiolipins (CLs), phosphatidylserines (PSs) and phosphatidylglycerols (PGs) (Fig. 2C-G). Sphingomyelins (SMs) have a different basic structure, but also contain two alkyl moieties (the sphingosine backbone and the *N*-acyl group), resulting in good linearity (Fig. 2H). Finally, although the most abundant phospholipid class, phosphatidylcholines (PCs), has two acyl groups and is extracted in the apolar phase, it has a lower *r*^2^ of 0.667 (Fig. 2I). This is due to its high abundance, saturating the detector at higher worm numbers. Indeed, when considering linearity for ≤4000 worms, the *r*^2^ for PC is 0.832 and even goes up to 0.902 when analyzing up to 2000 worms. It is therefore advised to use ≤4000 worms in order to accurately measure PCs. Phosphatidylethanolamines (PEs) show the same trend, although to a lesser extent (Fig. 2J).

At the other end of the lipid polarity spectrum are lysolipid species such as lysophosphatidylcholine (LPC), lysophosphatidic acid (LPA), lysophosphatidylethanolamine (LPE) and lysophosphatidylglycerol (LPG), each containing only a single fatty acid side chain (R_1_) and a polar head group. Similar to PCs, LPC abundance is high and its detection reaches a plateau at higher worm numbers (Fig. 2K). When including all data points, up to 8000 worms, the *r*^2^ is 0.430, but this improves (*r*^2^=0.872) when including ≤2000 worms. Other lysophospholipids are poorly detected in the chloroform phase of the current two-phase method, resulting in loss of linearity (Fig. S1A-C). Interestingly, despite containing two fatty acid side chains, phosphatidic acid (PA), and bis(monoacylglycero)phosphate (BMP) show *r*^2^s of 0.785 and 0.667, respectively (Fig. S1D,E). Owing to these complex lipid properties relating to solubility, it is likely that these lipids are (partly) extracted to the polar phase during the two-phase LLE. The final solvents of the metabolomics and lipidomics method are incompatible in the ultra-performance LC (UPLC), and none of these lipid species were measurable using the ZIC-cHILIC platform. However, we performed our two-phase extraction on 2000 control worms and analyzed both phases using the lipidomics solvents and analytical platform. When plotting the abundance of lysolipid internal standards in each of the layers compared to their non-lyso counterparts (Fig. S1F), we clearly observed that the LPG internal standard partitions almost completely to the polar layer. The internal standard of LPA, LPE and LPC also show partial solubility in the polar phase (Fig. S1F).

This effect is reflected in the RSDs when comparing the one-phase to the two-phase extraction. Using a one-phase extraction on five biological replicates, 85% of lipid species showed an RSD <30. When using the two-phase extraction on five biological replicates, 69% of detected lipid species showed an RSD <30. Repeated injections of a pooled sample of both of these experiments showed an RSD <30 in 94% lipid species for the one-phase method, while showing the same for 89% of species in the two-phase extraction (Table S3). To explore these partitioning effects on the detected lipidome in more detail, we made a direct comparison between the detected lipidome of the one-phase extraction (Gao et al., 2017) and the new two-phase extraction of samples containing ∼2000 worms from the exact same biological experiment. When plotting all the individual lipid species, we observed that, for most lipids, the measured abundance is highly similar between the one-phase and two-phase extraction (Fig. 2L), suggesting that there was no significant loss of these lipid species in the polar extraction phase. However, ∼9% of the species that are normally detected using the one-phase extraction are not detected when applying the two-phase method, most strikingly the entire LPE class (Fig. S1B). On the other hand, 10% of the lipids were only detected in the two-phase extraction, such as some BMPs and other low-abundant lipids (Fig. 2L). Possibly, these low-abundant lipids are not detected with the one-phase extraction owing to suppression effects in the MS. In conclusion, despite the loss of some polar lipid species, including the whole LPE class, there were some low-abundant lipid species only recovered using the two-phase extraction. Most importantly, the vast majority of major lipid classes are detected equally well with the two-phase extraction compared to the one-phase extraction.

### Polar and apolar metabolites change upon knockdown of metabolic genes in *C. elegans*

**Fig. 3.**
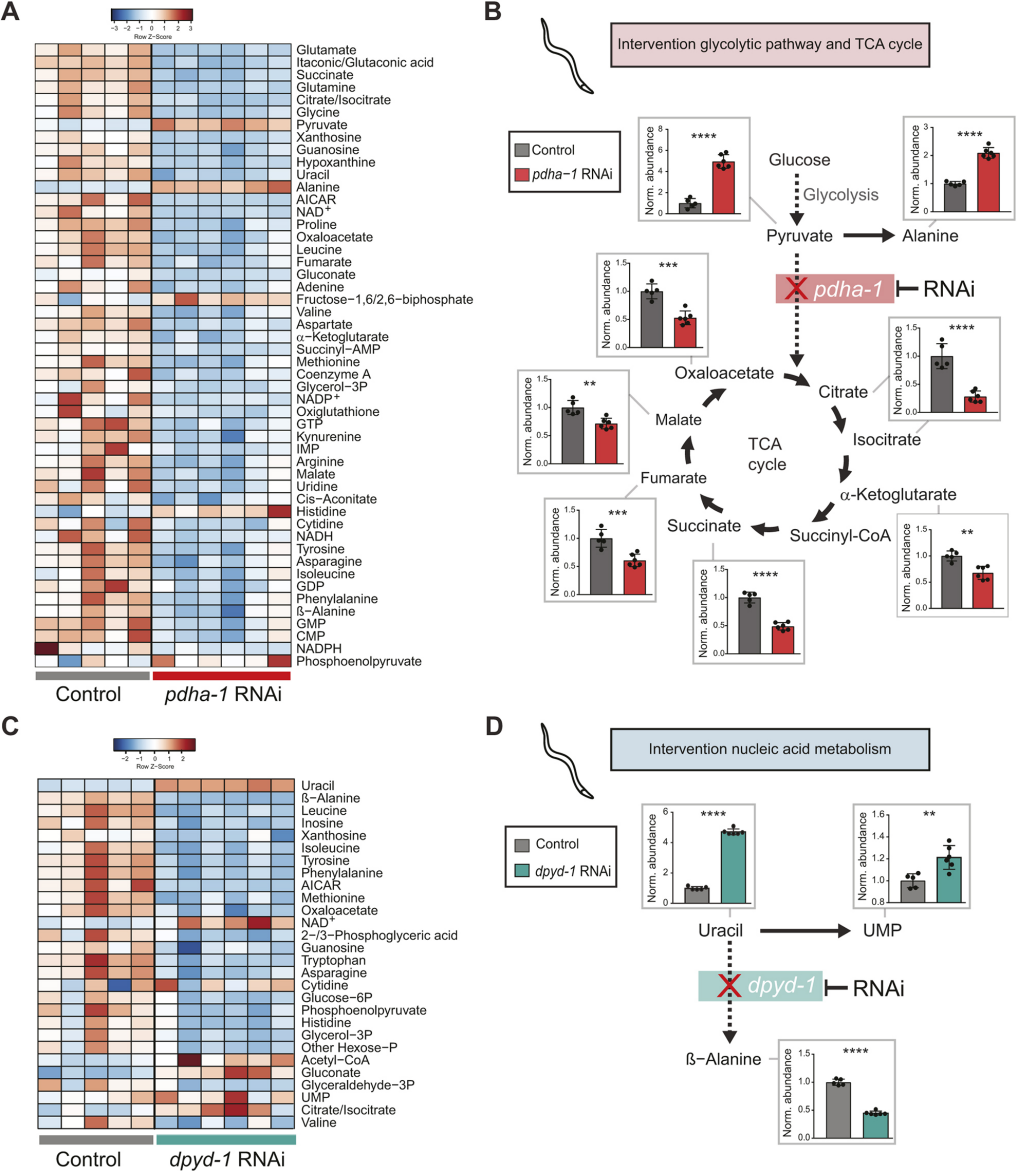
**Metabolite changes in worms treated with either *pdha-1* RNAi or *dpyd-1* RNAi.** (A) Heatmap of metabolite changes sorted on FDR of *pdha-1* RNAi-treated worms compared to control worms treated with an empty vector. (B) RNAi of the *pdha-1* enzyme metabolizing pyruvate into acetyl-CoA, providing the primary link between glycolysis and the tricarboxylic acid (TCA) cycle, results in significant increases in pyruvate and alanine and significant decreases in TCA cycle intermediates (iso)citrate, α-ketoglutarate, succinate, fumarate, malate and oxaloacetate. (C) Heatmap of metabolite changes sorted on variable importance in projection (VIP) score of *dpyd-1* RNAi-treated worms compared to control worms treated with an empty vector. (D) RNAi of the *dpyd-1* enzyme catalyzing uracil, which ultimately ends in β-alanine (via 5,6-dihydrouracil and N-carbamyl-β-alanine), results in significant upregulation of uracil and UMP and significant downregulation of β-alanine. Data points represent biological replicates with *n*=5-6, and significance (*P*<0.05) was tested with unpaired Student's *t*-test (**P<0.01, ***P<0.001, ****P<0.0001).

In order to test how well our method was able to pick up biologically relevant differences, we first targeted four different metabolic pathways using RNAi against enzymes or other factors known to affect metabolic pathways in *C. elegans* (Table S4). The first gene targeted was the pyruvate dehydrogenase alpha subunit (*pdha-1*), which is part of the complex that is responsible for converting pyruvate into acetyl-CoA. Because acetyl-CoA feeds into the tricarboxylic acid (TCA) cycle it links glycolysis to the TCA cycle, and RNAi of this enzyme is expected to affect both of these pathways. Indeed, worms treated with *pdha-1* RNAi show many significant changes in metabolite abundance (Fig. 3A). A fivefold increase was observed for pyruvate in these worms compared to worms treated with an empty vector, and a twofold increase was observed for alanine, which is an amino acid that can be formed from pyruvate (Fig. 3B). On the other hand, a significant decrease was observed for all TCA cycle intermediates we measured, including (iso)citrate, α-ketoglutarate, succinate, fumarate, malate and oxaloacetate (Fig. 3B). This is in line with a reduced availability of acetyl-CoA that can enter the TCA cycle.

The next enzyme we targeted was dihydropyrimidine dehydrogenase (*dpyd-1*), involved in pyrimidine base degeneration. DPYD-1 is important for nucleic acid metabolism as it catalyzes the reduction of uracil and is involved in the degradation of the chemotherapeutic drug 5-fluoroacil (5-FU). In this *dpyd-1* RNAi condition, we also found many significant metabolite changes compared to worms treated with empty vector (Fig. 3C). We observed an almost fivefold accumulation of uracil, accompanied by a small increase in uridine monophosphate (UMP), which can be alternatively metabolized from uracil (Fig. 3C,D). DPYD-1 is also involved in β-alanine biosynthesis (Wasternack, 1980). In line with this, a strong reduction in β-alanine was observed in the *dpyd-1* RNAi-treated worms (Fig. 3C,D), again reflecting the knockdown of this enzyme at the metabolite level.

**Fig. 4.**
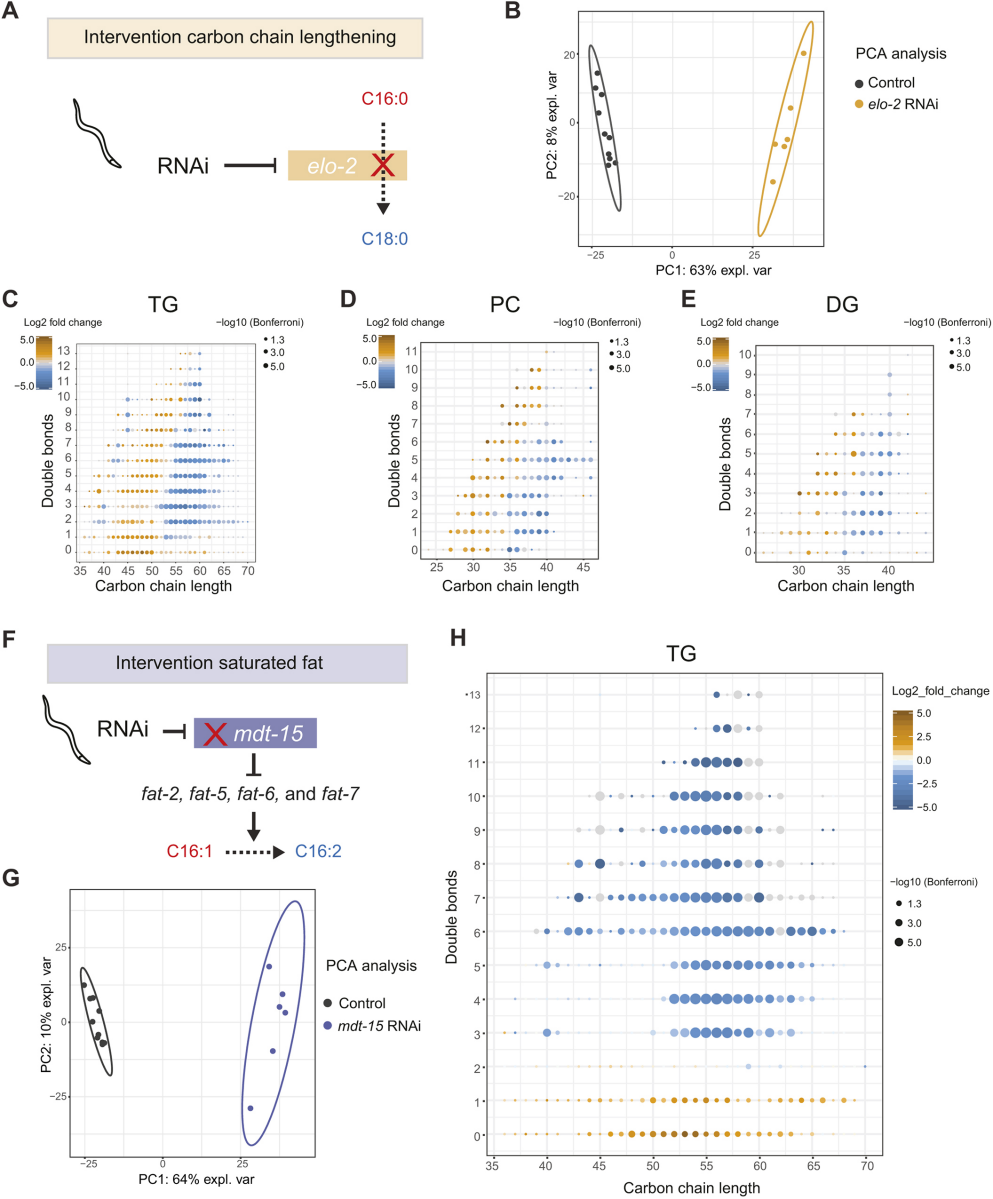
**Phospholipid changes in worms treated with either *elo-2* RNAi or *mdt-15* RNAi.** (A) RNAi of *elo-2*, which has fatty-acid elongase activity in worms, is expected to increase carbon-chain length of phospholipids. (B) PCA showing clear distinction between *elo-2* RNAi-treated worms and control worms (*n*=6 biological replicates). (C) Changes in the TG composition of *elo-2* RNAi versus empty vector controls shows significant decrease of phospholipids with long carbon-chain length (>55) and significant increase of TG species with short carbon-chain length (<55). (D,E) A similar pattern was observed in other phospholipid species such as PC (D) and DG (E). (F) RNAi of *mdt-15*, a transcription factor upregulating *fat-2*, *fat-5*, *fat-6* and *fat-7*, affects the level of unsaturation, i.e. carbon-chain double bonds. (G) PCA showing clear distinction between *mdt-15* RNAi-treated worms and control worms. (H) Changes in phospholipids of the TG species show significant decrease of lipids with >2 double bonds and increase of phospholipids with <2 double bonds.

We next set out to establish biological validation of our lipidomics analysis. With RNAi, we targeted an enzyme involved in fatty acid elongation, *elo-2* (Fig. 4A). When exploring the lipid profile of worms using our method, we could distinguish the control worms from the *elo-2* RNAi-treated worms, as shown with principal component analysis (PCA) (Fig. 4B). In order to visualize effects on lipid elongation, we then plotted carbon-chain length versus the total number of double bonds in those chains for individual lipid classes. For instance, TGs in *elo-2* RNAi-treated worms showed a marked decrease in lipids with long carbon chains and accumulation of lipids with shorter carbon chains regardless of the number of double bonds (Fig. 4C). The same was observed for PCs, DGs (Fig. 4D,E) and other lipid classes (Fig. S2). Our data confirm that elongation of carbon chains in fatty acids is inhibited when *elo-2* is knocked down in *C. elegans*, which leads to widespread changes across the lipidome.

Finally, we targeted *mdt-15*, a subunit of the Mediator complex. Rather than acting on fatty acid elongation, *mdt-15* transcriptionally regulates fatty acid desaturases including *fat-2*, *fat-5*, *fat-6* and *fat-7* (Fig. 4F) (Lee et al., 2015; Taubert et al., 2006). The PCA shows clear separation of the control worms from the *mdt-15* RNAi-treated worms based on their lipid profiles (Fig. 4G). When plotting carbon-chain length versus the number of double bonds, we observed a strong decrease in lipids with multiple double bonds and accumulation of lipids with ≤1 double bond, irrespective of the carbon-chain lengths (Fig. 4H; Fig. S3). This shift towards saturated TG species in worms treated with *mdt-15* RNAi is in line with the previously described regulation of *mdt-15* on fatty acid desaturation enzymes (Lee et al., 2015; Hou et al., 2014).

Together, these four different RNAi conditions affecting distinct metabolic pathways and metabolite classes illustrate that our method can adequately pick up relevant biological differences.

### Metabolic diversity in a *C. elegans* reference population

**Fig. 5.**
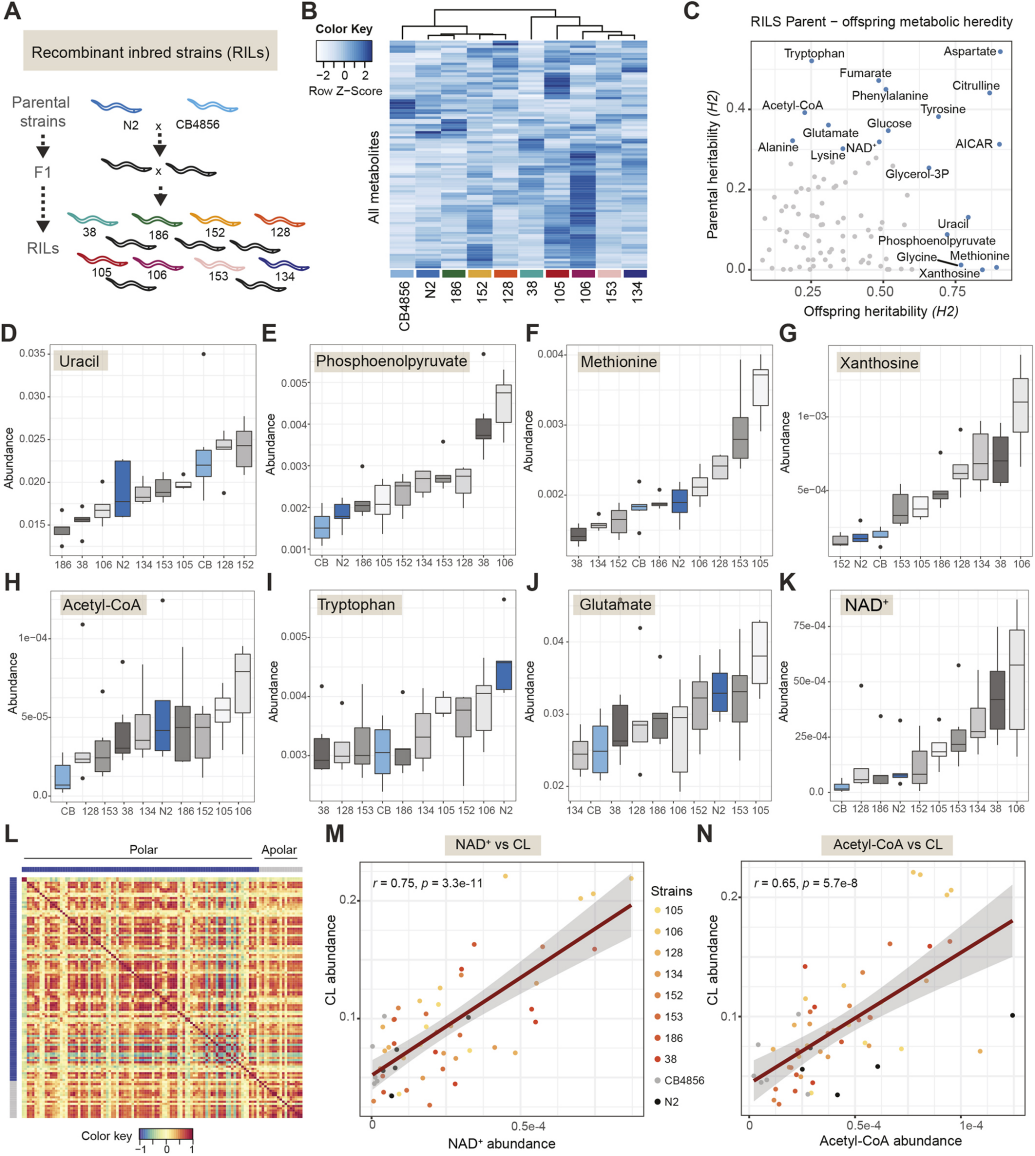
**Natural diversity of metabolite abundances in recombinant inbred lines (RILs).** (A) RIL strains selected for analysis. (B) Heatmap showing RILs and metabolites, showing diversity in metabolite levels present between the different strains (*n*=5-6). (C) Broad-sense heritability (*H*^2^) of offspring versus parental lines. Heritability indicates the percentage of variance for a given metabolite that is explained by genetics. Examples of diverse heritable outcomes include the following: (D) uracil (offspring *H*^2^ 0.794, FDR<0.05; parental *H*^2^ 0.131, FDR not significant); (E) phosphoenolpyruvate (offspring *H*^2^ 0.721, FDR<0.05; parental *H*^2^ 0.088, FDR not significant); (F) methionine (offspring *H*^2^ 0.892, FDR<0.05; parental *H*^2^ 0.006, FDR not significant); (G) xanthosine (offspring *H*^2^ 0.843, FDR<0.05; parental *H*^2^ 0.000, FDR not significant); (H) acetyl-CoA (offspring *H*^2^ 0.228, FDR not significant; parental *H*^2^ 0.392, FDR<0.05); (I) tryptophan (offspring *H*^2^ 0.252, FDR not significant; parental *H*^2^ 0.521, FDR<0.05); (J) glutamate (offspring *H*^2^ 0.310, FDR not significant; parental *H*^2^ 0.361, FDR<0.05); (K) NAD^+^ (offspring *H*^2^ 0.486, FDR not significant; parental *H*^2^ 0.319, FDR<0.05). (L) Cross-correlation matrix between polar (blue) metabolites and apolar (gray) lipid classes, highlighting the range of strong positive correlations (red) to strong negative correlations (blue) between all metabolites and lipid classes. (M) Example of correlation between apolar cardiolipins (CL) and polar NAD^+^ (Pearson's *r*=0.75, *P*=3.3×10^–11^); parental lines and strains are color coded as highlighted in the key. (N) Example of apolar CL and polar acetyl-CoA (Pearson's *r*=0.65, *P*=5.7×10^–8^).

Our method also allows for the exploration of the natural variation of metabolite abundances occurring due to the differences among genetic backgrounds. To demonstrate this, we turned to recombinant inbred lines (RILs) derived from wild-type worm strains N2 and CB4856 (Thompson et al., 2015; Li et al., 2006). RILs are genetic mosaics of the parental strains N2 and CB4856. We reasoned that the sensitivity of our approach could reveal the genome's more subtle influences owing to naturally occurring polymorphisms affecting the metabolome. Therefore, we proceeded to perform the current metabolomics method on two parental wild-type strains (N2 and CB4856) and eight different RIL strains resulting from the genetic cross (Fig. 5A; Table S5). Metabolic profiling revealed the underlying diversity of metabolites present in the different genetic backgrounds (Fig. 5B). To explore this in a more systematic manner, we calculated the broad-sense heritability (*H*^2^) for each metabolite, for both parental and offspring strains (Gao et al., 2018). *H*^2^ serves as an indication for the percentage of variance for a given metabolite that is explained by genetics. Plotting *H*^2^ for the parental versus offspring strains illustrates where new combinations of alleles may have severe effects on distinct metabolic profiles and thus indicate genetic complexity of the trait (Fig. 5C).

Assessing heritability in this manner, we observed metabolites for which parental strains possessed a low heritability score and their offspring possessed a high heritability score, indicating that there may be multiple loci of opposing effects regulating the metabolite's abundance. For example, uracil, phosphoenolpyruvate, methionine and xanthosine exhibited such patterns (Fig. 5D-G). Conversely, we also observed metabolites for which the parental strains possessed a high heritability score and the offspring possessed an equal or lower score, which likely indicates that there may be few loci, or even just a single locus, affecting the metabolite's abundance. Examples of these included acetyl-CoA, tryptophan, glutamate and NAD^+^ (Fig. 5H-K).

Performing metabolomics and lipidomics on the same samples enables data integration from both techniques. To illustrate, we performed cross-correlations between polar metabolites and lipid classes and visualized these in a correlation matrix (Fig. 5L). For example, we identified metabolites that correlated with CL, which is an important component of the inner mitochondrial membrane (Houtkooper and Vaz, 2008). We found that abundance of CL correlated significantly with NAD^+^ (Fig. 5M), which governs mitochondrial function through its role as an enzyme cofactor as well as being a substrate for sirtuins (Houtkooper et al., 2010a,b; Houtkooper et al., 2012). Likewise, CL significantly correlated with acetyl-CoA (Fig. 5N), which is in line with its role in acetyl-CoA synthesis (Raja et al., 2017). These correlations are an example of how metabolomics and lipidomics data can be integrated and explored to gain deeper insight into their cross-talk and inter-relations.

## DISCUSSION

Changes in metabolism are increasingly recognized as valuable markers of, as well as causal contributors to the development of, metabolic disease and aging. Increasingly comprehensive methods for the analysis of both polar (metabolomics) and apolar (lipidomics) metabolites have proven essential in these fields. Thus far however, these omics methods required dedicated sample preparation, and therefore a separate sample, for metabolomics and lipidomics. Here, we report a method that uses both the polar (metabolomics) and apolar (lipidomics) layer of a two-phase LLE and analyzes these using high-resolution MS methods, providing an elegant way of exploring a large range of the metabolome and lipidome in a single sample, covering >1100 annotated metabolites of different classes. We show here that this method is robust and sensitive enough to analyze a wide variety of metabolic pathways using both metabolomics and lipidomics, and capable of reliably pinpointing metabolic aberrations in these pathways.

Biologically relevant differences in central carbon as well as lipid pathways could be determined in detail. The effects of *pdha-1* inhibition using RNAi was reflected throughout the TCA cycle, and the inhibition caused a decrease in acetyl-CoA and an accumulation of its precursor, pyruvate. Additionally, the pyruvate accumulation was accompanied by a metabolic diversion, leading to higher alanine abundance and reduced levels of TCA intermediates. Together, this comprehensive profiling provides a more detailed picture of the metabolic state. For instance, changes in less directly related metabolites were also seen, such as the increase in histidine abundance. In this way, basic profiling of metabolic changes following *pdha-1* inhibition might uncover new (genetic) factors that contribute to metabolic adaptation in these circumstances. Such information is not only valuable for research in *C. elegans*, but could also be used to study processes in which pyruvate dehydrogenase is involved, such as the aberrant preferential activation of glycolysis in cancer cells (Kaplon et al., 2013) or the regulation of brown adipose tissue metabolism (Held et al., 2018). This is also the case for the metabolite changes observed upon *dpyd-1* RNAi in context to the chemotherapeutic drug 5-FU and, for instance, its toxicities in patients with DPYD variants (Sistonen et al., 2014).

Similarly, when knocking down *elo-2* using RNAi, robust changes were observed in the total chain length of almost all lipid classes. Knockdown of *mdt-15* led to significant changes in the degree of saturation of a wide variety of lipid species. Combined, this shows that our method provides an exceptionally detailed view on complex lipid composition, which can be useful for the identification and study of disorders related to the lipidome.

RILs were used to illustrate that our method is capable of picking up not just large metabolic defects caused by knockdown of a single gene, but also more subtle metabolic effects in a non-interventional population harboring genetic variation. Although our study is not large enough to parse out the complexity of the underlying genetic traits, we clearly show both convergent and divergent patterns of inheritance, as expected based on population genetics. Future studies using the full panel of RILs will allow the reconstruction of genetic complexity that causes individual metabolic variation and enable the study of gene-by-environment interactions, i.e. which genes render the organism susceptible to environmental disturbances.

We also used the RILs to highlight examples of direct integration of lipidomic and metabolomic data. These multi-omics comparisons are aided by using our method, as both datasets originate from the same material, eliminating the noise of inherent biological differences when independent worm cultures are prepared. Integrating metabolomics and lipidomics data can be important, as polar and lipid pathways are interconnected and often converge in meaningful ways. As an example, we showed that the mitochondrial lipid CL correlates with the abundance of polar metabolites with important roles in mitochondrial function such as NAD^+^ and acetyl-CoA across the RIL panel. Such integration based on population data can support new hypotheses and its validation in natural populations.

On the technical side, the use of a diverse selection of internal standards allows for meaningful semi-quantitative comparisons between sample groups. Owing to the ubiquitous and essential nature of many of the metabolites analyzed here, this dual extraction method can be developed to aid metabolomics and lipidomics in a wide variety of matrices. This is especially useful when the amount of available material is limited and two separate extractions might not be feasible, such as with human biopsy material or rare cell populations. When applying the current method to a new matrix, we strongly advise performing a range-finding experiment to determine a sample quantity in which most of the analytes of interest are in the linear range of the extraction and MS, as well as appropriate internal standards.

Although the aforementioned considerations are important for all metabolite extraction methods, one of the main limitations of this method is that some polar lipid species are not ending up in the apolar layer but in the polar layer and are therefore not measured in lipidomics. When specifically interested in such polar lipid species, a dedicated one-phase lipidomics extraction yields a better result (Gao et al., 2017). In conclusion, the currently presented method is capable of robustly analyzing a broad range of the metabolome and lipidome, and detecting biologically relevant differences while requiring only a single small sample.

## MATERIALS AND METHODS

### Worm growth conditions for RNAi experiments and RILs

Worm experiments were performed on independent biological replicates unless stated otherwise. N2 worms and RIL strains were cultured at 20°C on nematode growth medium (NGM) agar plates seeded with OP50 strain *Escherichia coli*. For RNAi knockdown experiments, we seeded 2000 synchronized eggs per 10 cm NGMi plate [containing 2 mM isopropyl β-D-1-thiogalactopyranoside (IPTG)] with a bacterial lawn of either *E. coli* HT115 (RNAi control strain, containing an empty vector) or *pdha-1*, *dpyd-1*, *elo-2* or *mdt-15* RNAi bacteria. Similarly, for the parental strains (N2 and CB4856) and eight different offspring (RILs strains WN038, WN105, WN106, WN128, WN134, WN152, WN153, WN186), 2000 synchronized eggs were seeded per 10 cm NGM plate with a bacterial lawn of *E. coli* OP50. After 48 h, the synchronous population at L4 larval stage was washed off the plates in M9 buffer, and the worm pellet was washed with dH_2_O three times and then collected in a 2 ml Eppendorf tube before being snap frozen and stored at −80°C. Worm pellets were freeze dried overnight and stored at room temperature until extraction.

### Two-phase extraction

In a 2 ml tube, the following amounts of internal standard dissolved in water were added to each sample of freeze-dried worms for metabolomics: adenosine-^15^N_5_-monophosphate (5 nmol), adenosine-^15^N_5_-triphosphate (5 nmol), ^13^C_6_-fructose-1,6-diphosphate (1 nmol), guanosine-^15^N_5_-monophosphate (5 nmol), guanosine-^15^N_5_-triphosphate (5 nmol), ^13^C_6_-glucose (10 nmol), ^13^C_6_-glucose-6-phosphate (1 nmol), ^13^C_3_-pyruvate (0.5 nmol). In the same 2 ml tube, the following amounts of internal standards dissolved in 1:1 (v/v) methanol:chloroform were added for lipidomics: BMP(14:0)_2_ (0.2 nmol), CL(14:0)_4_ (0.1 nmol), LPA(14:0) (0.1 nmol), LPC(14:0) (0.5 nmol), LPE(14:0) (0.1 nmol), LPG(14:0) (0.02 nmol), PA(14:0)_2_ (0.5 nmol), PC(14:0)_2_ (0.2 nmol), PE(14:0)_2_ (0.5 nmol), PG(14:0)_2_ (0.1 nmol), PS(14:0)_2_ (5 nmol), ceramide phosphocholine SM(d18:1/12:0) (2 nmol) (Avanti Polar Lipids, Alabaster, AL, USA).

After adding internal standard mixes, a 5 mm steel bead and polar phase solvents (for a total of 500 µl water and 500 µl MeOH) were added and samples were homogenized using a TissueLyser II (Qiagen) for 5 min at a frequency of 30 times/s. Chloroform was added for a total of 1 ml to each sample before thorough mixing. Samples were centrifuged for 10 min at 20,000 ***g***. Of the two-phase system that was now created with protein precipitate in the middle, the top layer containing the polar phase was transferred to a new 1.5 ml Eppendorf tube. The bottom layer, containing the apolar fraction, was transferred to a 4 ml glass vial. The protein pellet in between the two layers was dried and subsequently dissolved in 0.2 M NaOH for quantification using a Pierce™ BCA Protein Assay following the product protocol.

### One-phase lipidomic extraction

In a 2 ml tube, the following amounts of internal standards dissolved in 1:1 (v/v) methanol:chloroform were added to each sample: BMP(14:0)_2_ (0.2 nmol), CL(14:0)_4_ (0.1 nmol), LPA(14:0) (0.1 nmol), LPC(14:0) (0.5 nmol), LPE(14:0) (0.1 nmol), LPG(14:0) (0.02 nmol), PA(14:0)_2_ (0.5 nmol), PC(14:0)_2_ (0.2 nmol), PE(14:0)_2_ (0.5 nmol), PG(14:0)_2_ (0.1 nmol), PS(14:0)_2_ (5 nmol), ceramide phosphocholine SM(d18:1/12:0) (2 nmol) (Avanti Polar Lipid). After adding the internal standard mix, a steel bead and 1.5 ml 1:1 (v/v) methanol:chloroform were added to each sample. Samples were homogenized using a TissueLyser II (Qiagen) for 5min at 30 Hz. Each sample was then centrifuged for 10 min at 20,000 ***g***. The supernatant was transferred to a 4 ml glass vial.

### Metabolomics

After the polar phase was transferred to a new 1.5 ml tube, it was dried using a miVac vacuum concentrator at 60°C and processed as reported previously (Molenaars et al., 2020). The residue was dissolved in 100 µl 6:4 (v/v) methanol:water. Metabolites were analyzed using a Thermo Fisher Scientific Ultimate 3000 binary UPLC coupled to a Q Exactive Plus Orbitrap mass spectrometer. Nitrogen was used as the nebulizing gas. The spray voltage used was 2500 V, and the capillary temperature was 256°C. Other conditions were as follows: S-lens radio frequency (RF) level, 50; auxiliary gas, 11; auxiliary gas temperature, 300°C; sheath gas, 48; sweep cone gas, 2. Samples were kept at 12°C during analysis and 5 µl of each sample was injected. Injection order for samples was random, with an injection of a pooled sample every ten injections. Chromatographic separation was achieved using a Merck Millipore SeQuant ZIC-cHILIC column (PEEK 100×2.1 mm, 3 µm particle size). Column temperature was held at 30°C. Mobile phase consisted of (A) 1:9 (v/v) acetonitrile:water and (B) 9:1 (v/v) acetonitrile:water, both containing 5 mmol/l ammonium acetate. Using a flow rate of 0.25 ml/min, the LC gradient consisted of 100% B for 0-2 min, reach 0% B at 28 min, 0% B for 28-30 min, reach 100% B at 31 min, 100% B for 31-32 min. Column re-equilibration was achieved by increasing the flow rate to 0.4 ml/min at 100% B for 32-35 min. MS data were acquired using negative ionization in full-scan mode over the range of m/z 50-1200. Data were analyzed using Thermo Fisher Scientific Xcalibur software version 4.1.50. All reported metabolite intensities were normalized to appropriate internal standards, as well as total protein content in samples, determined using a Pierce™ BCA Protein Assay Kit. Metabolite identification has been based on a combination of accurate mass, (relative) retention times and fragmentation spectra, compared to the analysis of relevant standards. Metabolomics data from these experiments can be found in Tables S1, S2, S4 and S5, as well as in the MetaboLights online database under the following code: MTBLS2370 (https://www.ebi.ac.uk/metabolights/).

### Lipidomics

After the solvents containing the lipids were transferred to a 4 ml glass vial, they were evaporated under a stream of nitrogen at 45°C. The residue was dissolved in 150 μl of 1:1 (v/v) chloroform:methanol. Lipids were analyzed using a Thermo Fisher Scientific Ultimate 3000 binary UPLC coupled to a Q Exactive Plus Orbitrap mass spectrometer. Nitrogen was used as the nebulizing gas. The spray voltage used was 2500 V, and the capillary temperature was 256°C. Other conditions were as follows: S-lens RF level, 50; auxiliary gas, 11; auxiliary gas temperature, 300°C; sheath gas, 48; sweep cone gas, 2. For normal-phase separation, 2 μl of each sample was injected onto a Phenomenex^®^ LUNA silica, 250×2 mm, 5 µm 100 Å. Injection order for samples was random, with an injection of a pooled sample every ten injections. Column temperature was held at 25°C. Mobile phase consisted of (A) 85:15 (v/v) methanol:water containing 0.0125% formic acid and 3.35 mmol/l ammonia and (B) 97:3 (v/v) chloroform:methanol containing 0.0125% formic acid. Using a flow rate of 0.3 ml/min, the LC gradient consisted of 10% A for 0-1 min, reach 20% A at 4 min, reach 85% A at 12 min, reach 100% A at 12.1 min, 100% A for 12.1-14 min, reach 10% A at 14.1 min, 10% A for 14.1-15 min. For reversed-phase separation, 5 μl of each sample was injected onto a Waters HSS T3 column (150×2.1 mm, 1.8 μm particle size). Injection order for samples was random, with an injection of a pooled sample every ten injections. Column temperature was held at 60°C. Mobile phase consisted of (A) 4:6 (v/v) methanol:water and B 1:9 (v/v) methanol:isopropanol, both containing 0.1% formic acid and 10 mmol/l ammonia. Using a flow rate of 0.4 ml/min, the LC gradient consisted of 100% A at 0 min, reach 80% A at 1 min, reach 0% A at 16 min, 0% A for 16-20 min, reach 100% A at 20.1 min, 100% A for 20.1-21 min. MS data were acquired using negative and positive ionization using continuous scanning over the range of m/z 150-2000. Data were analyzed using an in-house developed metabolomics pipeline written in the R programming language (https://www.r-project.org/). In brief, it comprises the following five steps: (1) pre-processing using the R package XCMS; (2) identification of metabolites; (3) isotope correction; (4) normalization and scaling; and (5) statistical analysis (Herzog et al., 2016). All reported lipids were normalized to corresponding internal standards according to lipid class, as well as total protein content in samples, determined using a Pierce^TM^ BCA Protein Assay Kit. Lipid identification has been based on a combination of accurate mass, (relative) retention times and the injection of relevant standards. Lipidomics data from these experiments can be found in Tables S3-S5, as well as in the MetaboLights online database using the code MTBLS2370 (https://www.ebi.ac.uk/metabolights/).

### Heritability estimation of RILs and integration of metabolomics and lipidomics

*H*^2^ was calculated as described previously (Gao et al., 2018). Briefly, using an ANOVA explaining the metabolite variation over the offspring strains, *H*^2^ was calculated as *H*^2^=*V*_g_/(*V*_g_+*V*_e_), where *V*_g_ is the variance attributed to genetics and *V*_e_ is the variance attributed to other factors (e.g. measurement uncertainty or other biological factors). Significance of the heritability was calculated by permutation, where the trait values were randomly assigned to strains. Over these permutated values, the variance captured by strain and the residual variance were calculated. This procedure was repeated 1000 times for each trait. The obtained values were used as the by-chance distribution, and a false discovery rate (FDR) of 0.05 was taken as the 50th highest value. In the parental strains, *H*^2^ was calculated as *H*^2^=0.5*V*_g_/(0.5*V*_g_+*V*_e_). The factor 0.5 corrects for the overestimation of the additive variation in inbred strains (Hegmann and Possidente, 1981). The same permutation approach as for *H*^2^ was applied, taking the FDR=0.05 threshold as significant. An *H*^2^ above the FDR value indicates that there is only a 5% chance the result is a false positive.

Cross comparisons between metabolites and lipids were performed as follows: individual metabolite abundances were used. Lipid class abundances were calculated by summing the abundances of each lipid species from a given lipid class. These were cross-correlated using the imgCor function from the mixOmics package v6.6.2 (Rohart et al., 2017) in R. Association and significance between lipids and metabolites was tested for using Pearson's product moment correlation coefficient. Visualization of data was performed using ggplot2 (Wickham, 2009). R v3.4.3 and Bioconductor v3.5 were used in these analyses.

## Supplementary Material

Supplementary information
