# Supplementary material for: Metabolomics and lipidomics in Caenorhabditis elegans using a single-sample preparation
Source: Dis Model Mech. 2021 Apr 27;14(4):dmm047746. doi: 10.1242/dmm.047746 (PMC8106956; doi:10.1242/dmm.047746)
Supplement: Supplementary information [file dmm-14-047746-s1.pdf]

Figure S1

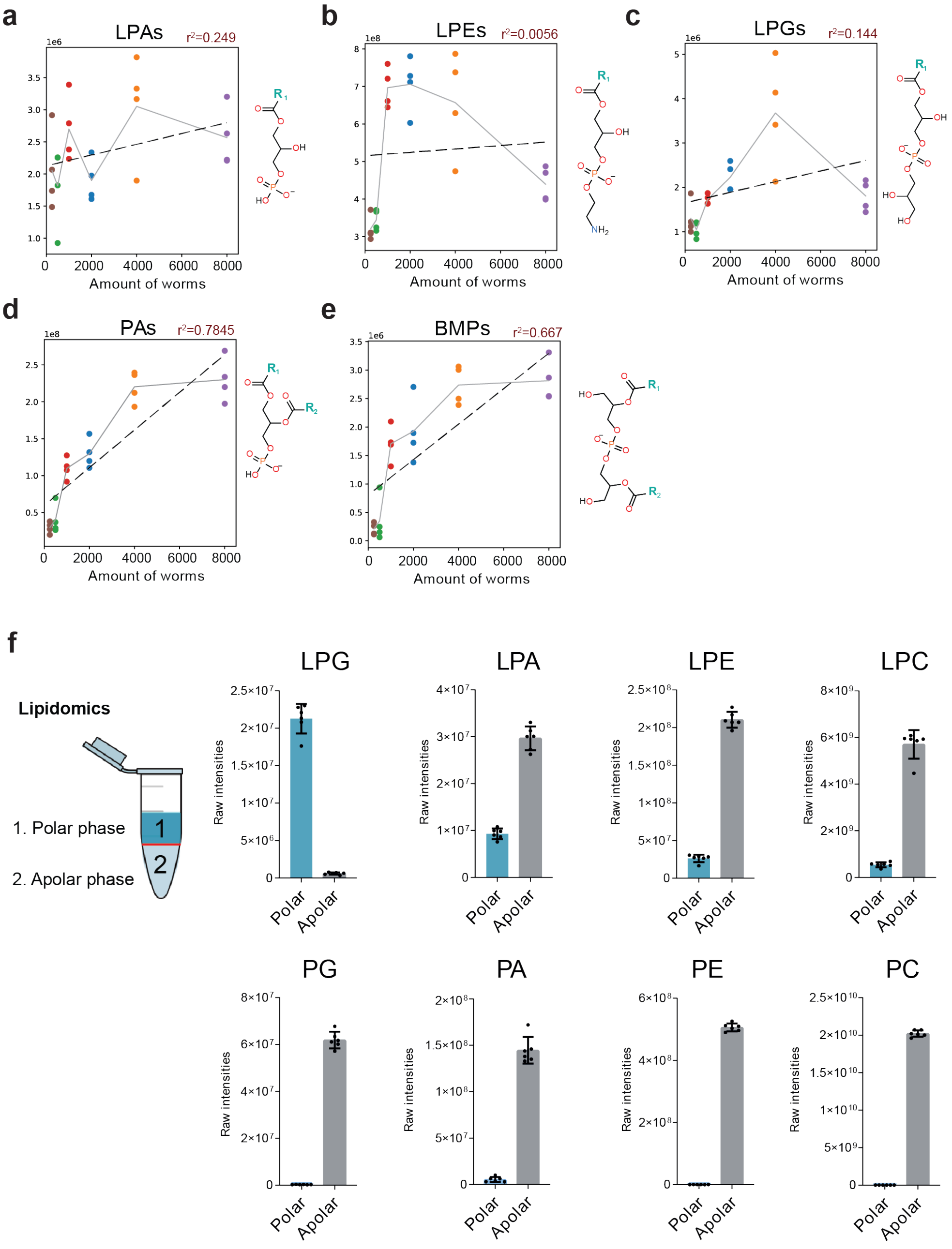

Fig. S1 | Validation of lipids extracted from apolar phase on the polar side of the lipid spectrum. Linearity of **a,** LPAs **b,** LPEs **c,** LPGs **d,** PAs **e,** BMPs. Chemical structure of the classes are depicted on the right of each graph. **f**, Partitioning of internal standards into apolar/polar phase. Internal standards of lyso-lipid species LPG, LPA, LPE, LPC partially go to the polar phase, while non-lyso counterparts stay in the apolar phase.

Figure S2

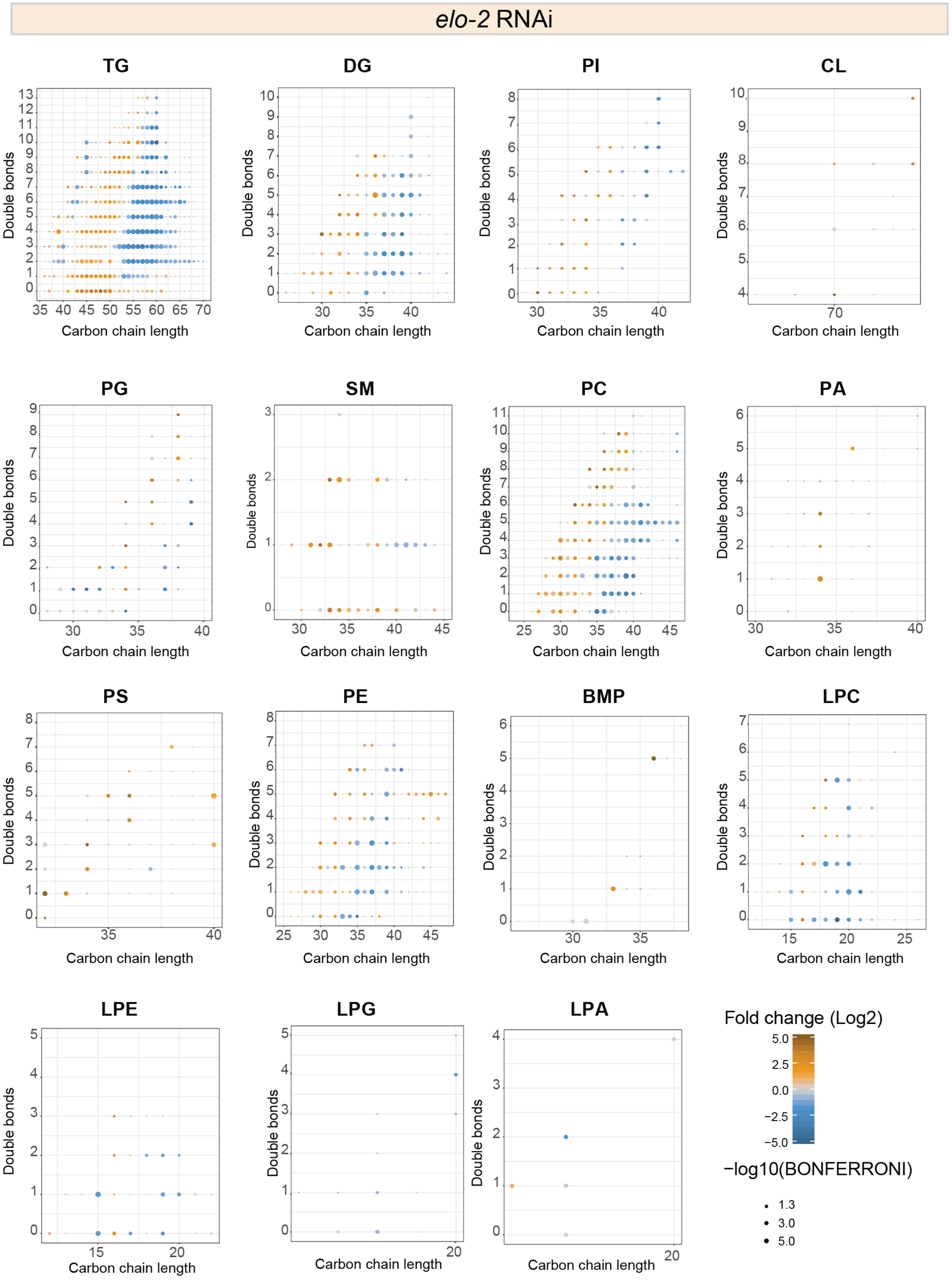

Fig. S2 | Phospholipid changes in all classes in worms treated with *elo-2* RNAi. Changes in the composition of almost all classes shows significant decrease of phospholipids with long carbon-chain length and significant increase of species with short carbon-chain length.

Disease Models & Mechanisms • Supplementary information

Figure S3

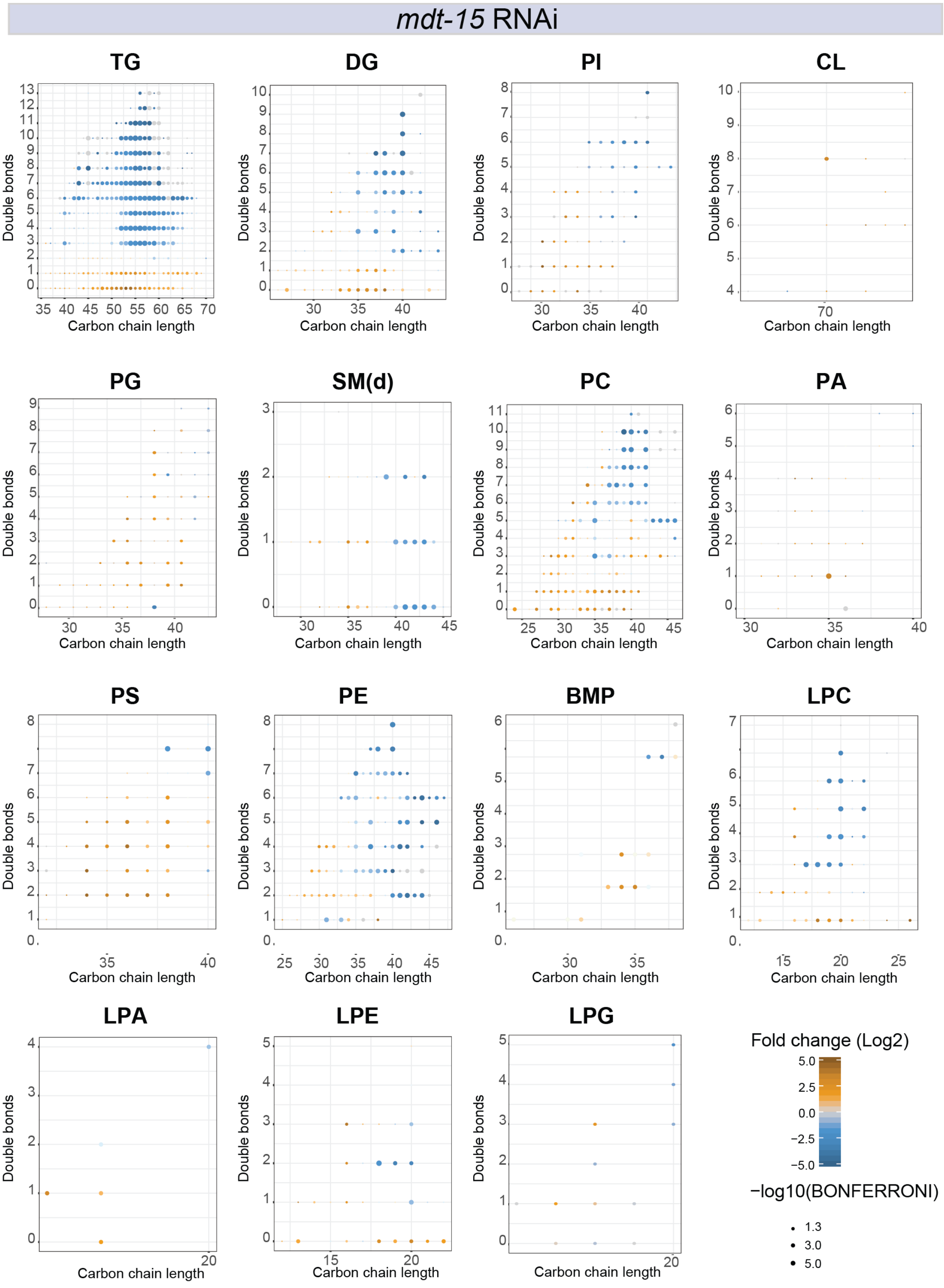

Fig. S3 | Phospholipid changes in all classes in worms treated with *mdt-15* RNAi. Changes in phospholipids of many species shows significant decrease of lipids with >2 double bonds and increase of PL with <2 double bonds.

Table S1. Data used to create Fig. 1B-E

Click here to Download Table S1

Table S2. Data used to create Fig. 1F-M

Click here to Download Table S2

Table S3. Data used to create Fig. 2

Click here to Download Table S3

Table S4. RNAi conditions used in Figs 3 and 4

Click here to Download Table S4

Table S5. Data used to create Fig. 5 (metabolomics and lipidomics data)

Click here to Download Table S5
